# Development of a Virtual Robot Rehabilitation Training System for Children with Cerebral Palsy: An Observational Study

**DOI:** 10.3390/s24248138

**Published:** 2024-12-20

**Authors:** Zhenli Lu, Yuming Luo, Marko Penčić, Dragana Oros, Maja Čavić, Verislav Đukić, Rastislava Krasnik, Aleksandra Mikov, Marko Orošnjak

**Affiliations:** 1 School of Electrical Engineering and Automation, Changshu Institute of Technology, Changshu 215500, China; 2 School of Electrical Engineering, Yancheng Institute of Technology, Yancheng 224007, China; 3 Faculty of Technical Sciences, University of Novi Sad, 21000 Novi Sad, Serbia; spawn@uns.ac.rs (D.O.); scomaja@uns.ac.rs (M.Č.); orosnjak@uns.ac.rs (M.O.); 4 Djukic Software GmbH, D-90408 Nürnberg, Germany; info@djukic-soft.com; 5 Faculty of Medicine, University of Novi Sad, 21000 Novi Sad, Serbia; rastislava.krasnik@mf.uns.ac.rs (R.K.); aleksandra.mikov@mf.uns.ac.rs (A.M.); 6 Clinic for Children Habilitation and Rehabilitation, Institute for Children and Youth Health Care of Vojvodina, 21000 Novi Sad, Serbia

**Keywords:** rehabilitation training system, virtual robot, children with cerebral palsy, therapy, human–robot interaction, social interaction, non-verbal communication, helpless shrug movement

## Abstract

This paper presents the development of a robotic system for the rehabilitation and quality of life improvement of children with cerebral palsy (CP). The system consists of four modules and is based on a virtual humanoid robot that is meant to motivate and encourage children in their rehabilitation programs. The efficiency of the developed system was tested on two children with CP. The effect of using the robot is an increase in the number of exercise repetitions, as well as the time spent on therapy, developing and strengthening the child’s musculature. Additionally, the children are able to produce socially acceptable gestures in the context of non-verbal communication for socialization. The main advantages of this system are its flexibility and ease of use. Besides the proposed use in CP rehabilitation, this system can be used in the rehabilitation of people recovering from surgery or injuries. Use of the proposed system significantly decreases the work load of the therapist who would be conducting the repetitive motion, allowing the therapist to see an increased number of patients. In the future, the number of different movements the robot is able to perform will be increased by way of domain-specific modelling and language.

## 1. Introduction

Cerebral palsy (CP) is considered to be one of the most common neurological disorders affecting children [1]. CP is a group of non-progressive but often variable motor function disorders caused by developmental issues or damage of the brain during the early stages of development [2]. Children with CP are characterized by a decreased range of motion of their joints, as well as the formation of contractures and deformities in and around their joints and bones, which most often affect the upper and lower extremities, as well as the spinal column [3]. CP can limit individuals in everyday activities and often comes hand in hand with visual and speech disorders, epilepsy, and hearing loss [4]. When determining the type of CP present, besides the medical history, clinical examination, and additional diagnostics, many different classification systems are used to evaluate the degree of disability according to gross and fine motor function, as well as speech [5,6,7]. According to the Surveillance of Cerebral Palsy in Europe (SCPE) database [8], the most common type of CP is the spastic type, with more than 80% of cases belonging to this type, with most of them being bilateral, and a smaller number classified as unilateral.

The level of physical activity of children with CP is lower than that of their peers, which makes it necessary to encourage them from a young age to be active in many different situations, where a level of motor function disability directly impacts their quality of life [9]. The level of fine motor function disability of children with CP aged 4–18 is assessed using the Manual Ability Classification System (MACS) [5]. To assess the level of gross motor function disability, the Gross Motor Function Classification System (GMFCS) [6] is used, with the first level being the least severe, in which children with CP can walk independently or with the use of orthosis, and the last level being the most severe, when the patient is mobile only with a wheelchair and the assistance of another person. To assess the speech-related level of disability, the Communication Function Classification System (CFCS) [7] is used, with the following classifications: (i) effective communication with familiar and unfamiliar partners, (ii) effective communication with familiar, but less effective with unfamiliar partners, (iii) effective communication with familiar, but not effective with unfamiliar partners, (iv) communication is sometimes effective with familiar partners, and (v) communication is seldom effective, even with familiar partners. These communication difficulties are key in the socialization of children with CP, since they severely affect their quality of life.

Keeping in mind the nature of CP and that there are no two children with identical clinical manifestations [10], it is key to notice the developmental deviation as early as possible and to begin rehabilitation. The main goal of therapy is to strengthen the child’s musculature [11], such as the torso, hips, and shoulders, to aid in upholding body posture, the ability to walk, and the ability to move their arms during everyday tasks, such as using eating utensils, brushing teeth, maintaining hygiene, etc. Another goal of therapy is the improvement of the child’s motor function, with the success rate depending directly on the child’s ability to perform the exercises at an appropriate level of intensity and number of repetitions. Accordingly, we propose a virtual robot rehabilitation training system for the improvement of the child’s cognitive abilities by way of fine motor function exercises, as well as their socio-emotional abilities by way of verbal and non-verbal communication, which is the goal of this paper.

Although the success of the therapy is directly proportional to the duration of the therapy—the time the child spends doing the exercises—executing the required motions can be an issue. Further, the motions—even the simplest ones—are often strenuous, painful, and tiring, so the child quickly loses interest in the exercise. Therefore, we propose a virtual robot which will partner with the children and will be able to demonstrate the exercises to make them more interesting. The virtual robot first establishes a connection with the child through verbal communication and then proceeds to demonstrate the appropriate exercise. After this, the child needs to repeat the exercise as many times as they can, and after every successful repetition, the virtual robot rewards the child with a non-verbal cue. This approach is expected to increase the success rate of therapy compared to that of the conventional approach.

The use of a physically present robot in the treatment of children with CP has positive effects on the children’s interest in the exercises, as the robot motivates and encourages them to exercise longer, when compared to the results for the conventional approach [12,13,14]. However, it is necessary to create an individualized exercise program, since different motor function and mental disabilities can present in children with CP. An additional issue is the necessary presence of an engineer during the whole therapy session (to activate the robot, to input different commands, etc.), as well as the impossibility of creating a specialized therapy scenario for each individual child. A virtual robot would potentially be very useful, since it would be much easier to create an individual therapy scenario using such a robot. The use of visual aids, computer simulations, and video games in the treatment of children with CP has been shown to be greatly motivational, increasing the therapy success rate [15,16,17].

Therefore, we propose a hybrid robot technology solution for the rehabilitation and improvement of the quality of life of children with CP. The structure of the paper is as follows: the first section describes the motivation and research goal; the second section presents the current state of the art; the third section explores the problems of this paper in detail; the fourth section shows the development and structure of the rehabilitation training system; the fifth section, based on an observational study, determines the efficiency of the proposed rehabilitation training system; and finally, the sixth section contains a discussion of the results, the conclusions, and the possibilities for future research.

## 2. State of the Art

A review of the available literature encompassed two groups: (i) the application of robots and exoskeletons in the re/habilitation of children with CP, and (ii) virtual robot platforms and systems for motion detection and recognition used in re/habilitation. Within the first group, most of the reviewed systems involved the use of a robot arm—an exoskeleton—as a support for patients performing physical exercises as part of their physical therapy. The second group covers different types of systems for motion detection and recognition (3D cameras, inertial measuring unit (IMU) sensors, biosensors, the Kinect system, etc.), as well as the design of virtual reality systems (virtual robots and game-like applications).

### 2.1. Robots and Exoskeletons in the Rehabilitation of Children with CP

The Armeo^®^ Spring exoskeleton with five degrees of freedom (DOFs) used in the re/habilitation of children with CP and acquired brain injury is shown in Ref. [18]. This exoskeleton offers passive arm-weight support by using a system of springs; sensors built into the exoskeleton allow for the detection and quality evaluation of the child’s arm movement. The exoskeleton is also connected to a virtual exercise system to create a feedback loop during the therapy.

The paediatric robotic arm ChARMin, with a total of four DOFs, meant for the re/habilitation of the upper extremities of children with damaged motor function, is shown in Ref. [19]. Based on the level of damage, the kinematic parameters of the robot can be adjusted; also, the robot elbow joint can be remotely activated to support the patient. The sensor information is forwarded to a computer (virtual model) that displays the motion in real time.

A motor learning method for the habilitation of children with hemiplegic CP is shown in Ref. [20]. An analysis was conducted before and after a training session on the movements of children with hemiplegic CP, aged 5 to 12. The training session consisted of point-to-point arm movements, with and without robot assistance. The results showed a marked improvement with the use of robot assistance, specifically in line following, point targeting, and the speed and ease of movement of the child’s arm.

A haptic robotic system with complex virtual environments meant to treat impaired upper extremity motor function in children with CP is shown in Ref. [21]; a HapticMaster robot with six DOFs includes 3D sensors to measure the position, velocity, and force (that the user applies to the robot) in real time; using the proposed system, experiments were conducted to determine the extent to which the robot eases the execution of the complex movements of children; the patients were required to show motion precision and repeatability during their training sessions, through game-like virtual simulations. It should be noted that the use of robots aided the improvement of motor skills in children with CP.

An artificial neuromolecular (ANM) system for robotic arm motion control meant for rehabilitation is shown in Ref. [22]. A virtual robot experimental platform was built for the realization and testing of the ANM system; this system was developed to improve issues such as joint rotation angle and start-up time through autonomous learning.

Upper limb exercises that use physical and virtual robots are shown in Ref. [23]. The experimental method is based on monitoring and comparing the relationship between sensory sensitivity and user performance when completing a task with a real or a virtual robot. The R1 robot has arms with a total of eight DOFs, and mounted on the head is a red-green-blue-depth (RGB-D) camera; after processing the data from the camera, the robot provides appropriate audio feedback to the user. It was concluded that the users responded more favourably to the real robot compared to the virtual robot.

### 2.2. Virtual Robots and Systems for Movement Detection and Recognition in Rehabilitation

A virtual reality (VR) robot for the re/habilitation of children with CP is shown in Ref. [24]. The authors designed a VR game to motivate the children to complete tasks during physical therapy; the range, precision, and number of repetitions of the motion are recorded so that the level of success can be assessed automatically. The game consists of a VR playground with a virtual robot, and the robot leads the children to different objects which they then need to attempt to pick up. The advantage of the VR robot is that it reduces both time and financial costs, while the VR game was both interesting and fun to the children.

A new system for emotion recognition based on tracking human movements is shown in Ref. [25]. To detect the movement, the authors placed wearable IMU sensors, i.e., a magnetometer, gyroscope, and accelerometer on the wrists, waist, and head of the test subject, respectively. A module was developed to collect the signal from the IMU sensors, and then an attention-based sensor fusion module was produced to process the signals after their collected. To increase the precision of emotion recognition, a weighted kernel support vector machine model was developed as well.

A system for emotion recognition of patients with mild cognitive impairment is shown in Ref. [26]. The socially-assistive robot NAO was used as an experimental platform, together with a rehabilitation program and a facial movement detection and recognition system; the software reliably detected seven facial expressions.

The design of a domain-specified language mechanism (DSLM)-based CP action rehabilitation training system is shown in Ref. [27]; the proposed hardware and software system enables upper extremity re/habilitation in children with CP by way of a virtual robotic system. A Kinect system was used to record the patients’ movements in real time; by following the movements of the virtual robot, the patients completed their physical therapy exercises in a fun way.

Robust hand gesture identification using an envelope of a high density-surface electromyography (HD-sEMG) signal is shown in Ref. [28]. These signals are recorded by a two-dimensional array of closely situated HD-sEMG electrodes which significantly increased the size of the database; the gestures of the test subjects were recognized by a support vector machine classifier.

A prototype 3D gaze-based robotic grasp that mimics human visuomotor function for people with motion impairments is shown in Ref. [29]. The authors developed a gaze vector model to analyse the eye movements of the patient; this model is used by patients with physical disabilities to control a robot arm when performing complex tasks.

A prototype of real-time brain machine interaction (BMI) via social robot gesture control is shown in Ref. [30]. The brain computer interface is based on neurofeedback and is proposed for use in personalized social robots for the rehabilitation of patients with cognitive impairments. An encephalogram was used to record the brainwaves of a patient attempting to control a mouse cursor on a screen, and the data were then further processed with software; this way, the patient could, in real time, use their thoughts to communicate with the robot by way of the developed BMI prototype, which then reacts to the actions the patient imagined.

Research on the simulated activities of daily living (ADL) tasks, using a vision-guided assistive robot arm, is shown in Ref. [31]. For the purpose of this experiment, a University of Central Florida (UCF)-ARM six DOFs assistive robot was used, together with a wheelchair-mounted robotic arm (WMRA) and an Exact Dynamics’ Manus ARM gripper with a built-in camera. In the experiment, healthy subjects had to complete the task of gripping an object at two different heights (floor and table), as quickly as possible and with the least amount of cognitive burden possible; it was concluded that this system can be used by patients with physical disabilities.

A prototype of an interactive socially-assistive 13 DOFs robot for patient care, for use in hospitals and elder care homes, is shown in Ref. [32]. The robot includes a vision system that recognizes gestures. To non-verbally interact with patients, the robot shows emotions by using its arms and characteristic parts of the face.

A data quantitative evaluation of the Microsoft KinectTM for use in an upper extremity virtual rehabilitation environment is shown in Ref. [33]. By using a Vicon Motion Capture system, the authors confirmed the classifications of the angles of the arm and shoulder during motion in space, specifically the shoulder abduction/adduction angle, shoulder flexion angle, and three-dimensional shoulder joint angle; furthermore, the Kinect system confirmed the quantitative measurement accuracy of these angles.

The development of a home-based VR therapy system to promote upper extremity movement for children with hemiplegic cerebral palsy is shown in Ref. [34]. The patients used the Sony PlayStation 2 with a camera to complete their VR therapy in a home environment, where they practiced arm (elbow and shoulder) and hand motion by playing games in a virtual environment; this system was proposed as a way to practice proximal movements of the hemiplegic hand/arm in a fun way.

Technologies facilitating home and community-based stroke rehabilitation are shown in Ref. [35]. The most often used community technologies, e.g., those used in rehabilitation centres, community hospitals, and at home, without a supervisor, as well as those used in remote rehabilitation, i.e., with a therapist, include video calls, voice communication, computer vision and sensing techniques, teleoperation, augmented reality (AR) and VR, robot-assisted methods, and a mobile remote presence. Within the bounds of community and at-home rehabilitation, the following were analysed: speech rehabilitation, physical rehabilitation, and total rehabilitation progress of the patient, while for remote rehabilitation, the authors analysed the diagnostic assessment of the patient, therapist intervention, and patient progress monitoring.

Biosignal-based human–machine interfaces (HMI) for assistance and rehabilitation are shown in Ref. [36]. Four categories of biosignals were considered as HMI control signals for prosthesis control, robot control, motion recognition, communication, and VR; possible uses for these interfaces would be with patients with muscular dystrophy, multiple sclerosis, CP, spinal cord injury, etc.

Wearable triboelectric devices for haptic perception and VR/AR applications are shown in Ref. [37]. These devices exhibit many valuable characteristics, such as small mass, high flexibility and sensitivity, and the possibility of both kinaesthetic and tactile perception. By way of artificial intelligence and machine learning, these devices can recognize complex motions performed by the robot, as well as human–machine interactions, which can greatly improve rehabilitation systems that include a physical or virtual robot.

A wireless inertial measuring system for human motion analysis is shown in Ref. [38]. The system consists of measuring units (a sensor network), an algorithm to determine orientation, and a VR platform; the measuring units are placed on the joints of the user, and they forward information to a computer in real time by way of wireless technology. The received information about the orientation and movement of the user are imported into a virtual robot platform to show the motion of the user; the suggested system is used to analyse gait and to gain visual information regarding the rehabilitation process.

### 2.3. Summary

Based on the available literature, it is concluded that different types of hardware (physically present robots and exoskeletons) and software (platforms in the form of virtual robots or other virtual systems) have been used with more/less success in the rehabilitation of patients with motor or cognitive disabilities. Also, the use of different non-verbal communication systems enabled the development of gesture databases for non-verbal communication between robots and patients during rehabilitation. It should be noted that patients reacted favourably to the use of virtual/real robots during rehabilitation. The platform that we propose is meant to address the requirements for the re/habilitation of children with CP with motor and/or cognitive disabilities in order to improve the motor, communication, and cognitive abilities of the child in everyday life activities. Detection of a helpless shrugging movement, as a spontaneous and non-verbal reaction of children with CP, using the proposed hardware, with real-time data processing and a display of the results, allows the therapist to monitor the success of the therapy and if necessary, immediately begin corrections of the child’s movements using functional tests.

## 3. Problem Description

Problems that occur in children with spastic CP can be motor function related, sensory, cognitive, intrapersonal, and interpersonal [39], and can impact their ability to care for themselves, to play, to perform different activities, etc. The ability to perform these activities positively impacts the health and quality of life of children with CP. Some of these activities include recreational physical activities, attending class, spending time with their families, taking care of themselves, etc.

Due to motor function disabilities, as well as the problems that go along with these, children and adolescents with CP often fall below the level of their peers with regard to motor skills. Children with CP often have issues with completing everyday tasks, especially tasks related to taking care of themselves, such as brushing their teeth and hair, dressing and undressing, using eating utensils, etc. Children with spastic CP, even when they are not intellectually disabled and have the motivation to complete these activities, their increased muscle tone impairs their fine and gross motor function, which infringes upon their ability to perform the aforementioned tasks.

Learning different ways to communicate and interact with others is of great importance for children with CP, specifically so they can mature and integrate into social groups. In this context, the shoulder shrugging movement, which is crucial in this research, represents a spontaneous non-verbal body reaction during human interaction. By forming appropriate social scenarios, it is possible to improve the communication and cognitive skills of children with CP. The expected effects of this kind of therapy are overcoming the physical barrier of movement and mastering body language needed for the socialization of children with CP.

Many different therapy modalities are used in the rehabilitation of children with CP, but the base of most of them is therapy done through movement—kinesitherapy—as well as occupational therapy. The treatment plan is on a child-to-child basis, adapted to both short- and long-term therapy goals, as well as to the clinical state, functional ability, and needs of the child in question. It is often conducted daily, over a long period of time. However, the success rate is largely dependent on the amount of time the child spends exercising, and the movements, even the simplest ones, are often strenuous, painful, and tiring, which causes the child to quickly lose interest.

While using the physically present robot MARKO [40] during the rehabilitation therapy of children with CP at the Clinic for the Habilitation and Rehabilitation of Children, Institute of Child and Youth Health, Vojvodina Novi Sad, Serbia, the following positive effects were noticed: improvement of active, wilful mobility in all of the upper limb segments; improvement of bimanual activities, muscle strength, movement selectivity, visual-motor control, and precision of movement, as well as an increase in the children’s endurance compared to the results for the conventional approach. On the other hand, the following issues were noticed: the necessary presence of an engineer during the whole therapy session to monitor the robot and to input commands; increased difficulty in the monitoring of the kinesitherapy process, especially in the case of technical issues with the robot; and the most glaring issue, the inability to adapt the therapy to each individual child.

### Summary

Considering a decade of experience in the development of socially assistive robots, as well as their application in the re/habilitation of children with CP, it was concluded that for each child, an appropriate therapy scenario would need to be created by reprogramming the physically present robot; personalizing therapy should increase the success of re/habilitation. We believe that a virtual robot would be of great benefit, as it would make it easier to create an individual physical therapy program. The virtual robot also offers the possibility of performing more complex exercises, depending on the motor function and mental status of the children undergoing therapy. With a virtual robot, the engineer’s presence is no longer necessary, increasing the user’s autonomy, which was a limiting factor when scheduling individual therapy sessions. The proposed hybrid therapy, based on a virtual robot, would encourage the child, in an interactive and fun way, to perform a specific kind of movement or exercise, activating targeted muscle groups, which would increase the success rate of the therapy, which is the main goal of this paper. With the goal of improving the cognitive and socio-emotional abilities of children with CP, we will consider the use of non-verbal communication during physical therapy, as well as its implications, focusing on body language involving the use of the arms and shoulders.

## 4. Novel Rehabilitation Training System

Within this section, functional tests were conducted to obtain the reference movement, as well as the position of the joints during the adopted movement, which are the input parameters for the development of the rehabilitation training system. Afterwards, the architecture and technical specifications of the proposed system are shown, as well as the operation principles of the patient movement detection application. Finally, the appearance and operation principles of the entire rehabilitation training system are shown, as well as the modules it comprises.

### 4.1. Reference Movement and Joint Positions

A generic gesture method as a novel tool in the design of social robots is presented in Ref. [41]. By generating a set of gestures for different morphologies, the importance of specific joints and their influence on a series of postures and gestures can be studied. The analysis of shoulder shrugging as a signal of hesitation or uncertainty in intercultural communication is presented in Ref. [42]; shrugging as a sign of hesitation is more often used in Western than in Eastern cultures. A small study was conducted regarding the differences in the interpretation of gestures from the video tape, after which conclusions were drawn on how these affect communication between different cultures; the aim of the study was for participants in the conversation to connect body movements with their meaning. The design and observed effects of robot-performed manual gestures is presented in Ref. [43]; the aims of the paper are to describe the gesture production process of a social robot and to provide a survey of the effects of robot-performed gestures on human–robot interactions. Unlike robots, people gesture spontaneously while speaking, and hand and face gestures have become an important feature of social robots. The effects of a robot’s use of hand gestures were analysed according to engagement, communicative purposes, task performance, perception of the robot, and aid provided to people with special needs.

During interpersonal communication, to supplement linguistic information, the shoulder shrugging motion often occurs as a spontaneous, natural reaction. This non-verbal answer to a posed question, with the meaning “I don’t know” or “I don’t understand the question”, is characterised by a specific position of the upper extremities—arms along the body, forearms approximately horizontal, with the hands open towards the other person and the shoulders raised. Although the shrug is most often accompanied by facial expressions—raised eyebrows and forehead, eyes open wider and lips in a different position [44], later in the paper, the focus will be solely on the movement of the upper extremities. To obtain the reference movement that occurs when shrugging, as well as to define the positions of the joints, two tests were conducted, with 15 healthy subjects each. It should be noted that the second test was a control test.

Test 1—The subjects were tasked with solving a Rubik’s cube in less than 1 min, after which the researcher asked each subject the control questions. The goal of the first test is to observe the non-verbal communication exhibited using the arms and shoulders. The subjects were instructed to answer the control questions verbally, while the researcher observes the non-verbal cues they express while answering. Each subject stood facing the researcher, 2 m away. The test was conducted under laboratory conditions, with controlled lighting and without any distractions to the subject. All of the subjects were students of the Changshu Institute of Technology in China. The subjects were not cognizant of the goal of the research and did not have any experience with similar tests. After obtaining their consent and familiarizing them with the task, the testing begun.

The researcher asks the first question (Q1): “Can you solve the Rubik’s cube within the allotted time?” After attempting to solve the cube, the subject answers the question. If the attempt was unsuccessful, the subject gives the verbal answer: “I didn’t succeed”. And at the same time spontaneously answers non-verbally with their arms and shoulders. Afterwards, the researcher asks the second question (Q2): “Do you think that I could solve the cube in the allotted time?” The subject answers verbally: “No, I don’t think that is possible”, while at the same time moving their arms and shoulders into the appropriate position. The researcher then proceeds to solve the cube within the allotted time and asks the third question (Q3): “I did it! Do you see?”, and the subject answers: “Congratulations, I can’t believe it!”, while gesticulating with their arms and shoulders.

**Figure 1:**
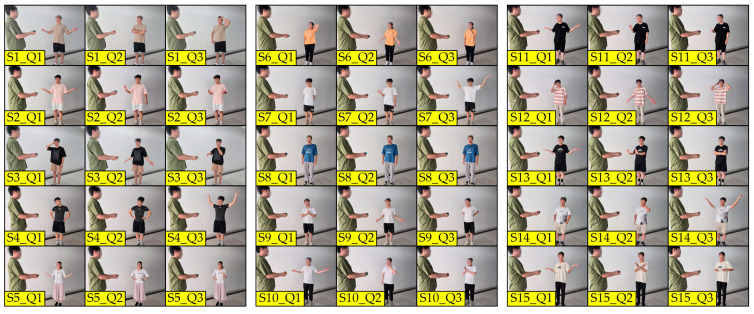
The first test results differentiated by the context of the verbal/non-verbal answer of the subjects: helplessness—column Q1, negation—column Q2, and surprise—column Q3.

Figure 1 shows all of the 15 subjects, as well as their non-verbal answers to the three questions, in the first, second, and third columns, respectively. Based on the analysis of the non-verbal answers, three types of shrugging motions can be differentiated according to the context: helplessness—column Q1, negation—column Q2, and surprise—column Q3. Table 1 shows the results of the first test based on the context of the verbal and non-verbal answer of the subject. All of the subjects provided verbal answers, while one subject did not provide any non-verbal answers. On the other hand, from the possible 45 non-verbal answers, only 14 were expressed, which is 31.1%. The highest number of subjects used the helpless shrug movement, specifically 46.7%. Negation was expressed with a shoulder shrug by 26.7% of the subjects, while only 20% of the subjects expressed surprise by shrugging their shoulders. Aside from this, it was ascertained by this test that the subjects take up slightly different positions (elbow height and hand position) for the same non-verbal answer, which is of importance for further analysis.

**Figure 2:**
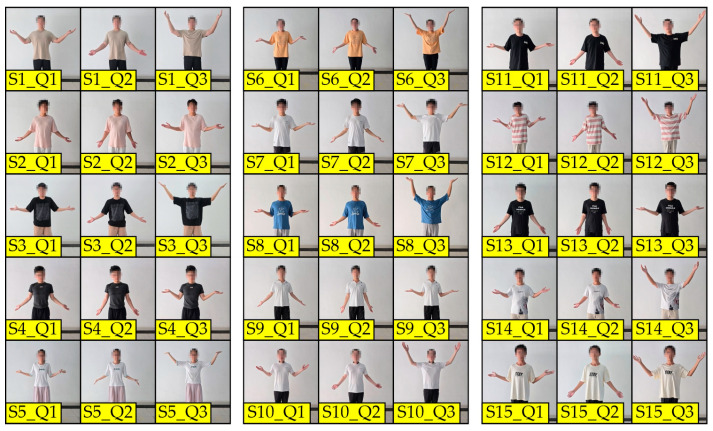
The results of the second test depending on the non-verbal context expressed by the subjects: helplessness—column Q1, negation—column Q2, and surprise—column Q3.

Test 2—The second test was designed to define the position of the shoulders, arms, and hands during the proposed movement and its context. Another goal of the second test is to confirm/refute the first test, specifically to determine which non-verbal context is most dominant with regard to spontaneity and how natural the motion is. The subjects were tasked with shrugging their shoulders to express different non-verbal contexts—negation, helplessness, and surprise—as shown in Figure 2. When conducting these voluntary movements, it was concluded that four subjects did not use their arms and shoulders significantly enough when expressing any of the three non-verbal contexts; thus, they could not be categorized. The remaining 11 subjects positioned their arms in ways they consider to be most expressive for each of the three non-verbal contexts.

**Figure 3:**
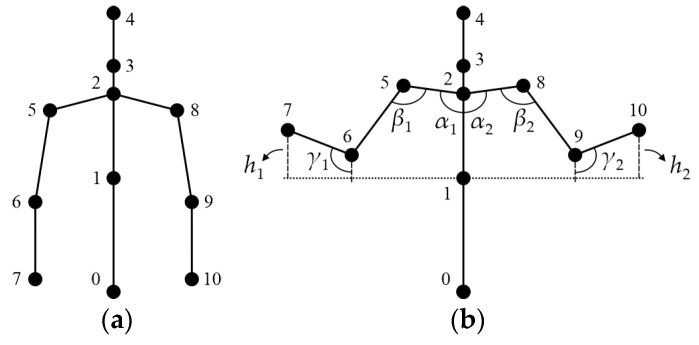
Simplified kinematic structure of the human upper body: (**a**) initial position; (**b**) helpless shrug position. Note: numbers 0 to 10 represent joints.

After analysing the position of the arms, hands, and shoulders while executing shrugs in all three non-verbal contexts, as well as reviewing the video footage, it was concluded that the joint positions of most of the subjects were close to each other for the helpless shrug movement, with the body moving the most naturally and with the most coordination. Further analysis showed that the subjects were not sure how to position their arms, hands, and shoulders when asked to express the other two non-verbal contexts—negation and surprise—which made these movements rigid and uncoordinated, which can easily be seen in the video footage as well. Accordingly, the helpless shrug was adopted as the reference movement for the remainder of the research, with the arms along the body, the forearms horizontal, the hands open, and the shoulders slightly raised. Finally, five of the subjects generated this movement again for the purpose of defining the reference positions of the shoulder, elbow, and wrist joints, which was then used to develop and calibrate the movement detection and identification system, according to Figure 3b. Table 2 shows the reference positions of the shoulder and arm joints of the subjects.

#### Summary

Functional tests were used to determine the reference movement as an essential input parameter for the development of the rehabilitation training system. According to the therapist’s recommendations, shrugging movements were analysed in test 1 for three different non-verbal contexts: negation, helplessness, and surprise. These shrugging movements were chosen because they are very often used in social interaction. Also, these movements and their contexts have similar joint positions during non-verbal communication using arms and shoulders. Functional test 1 determined the positions of the hands and shoulders when expressing spontaneous non-verbal responses to three verbal questions. Functional test 2 confirmed the results of the previous test, where the subjects voluntarily move their arms and shoulders to the positions they thought best represented all three non-verbal contexts. Based on the conducted tests and the analysis of the results, the helplessness shrugging movement was chosen as the dominant and reference movement due to the fact that the other non-verbal contexts were manifested to a much lesser extent.

### 4.2. System Architecture and Technical Specification

The rehabilitation training system consists of a Microsoft Kinect V2 device and appropriate software installed on a laptop computer, i.e., Open-Source Computer Vision Library—OpenCV v2.4.3, Visual Studio 2015 (VS2015), and CoppeliaSim 4.0 Edu.

#### 4.2.1. Development of the Motion Detection Environment

Microsoft Kinect V2 is an image capture and processing device that is able to 3D map and recognize human body movement and position, as well as open-hand and closed-hand gestures. This sensor system consists of a Microsoft HD colour camera that records data in a 1920 × 1080 px format, a time of flight (ToF) depth sensor with 30 frames per second (FPS), a Microsoft infrared camera with a resolution of 512 × 424 px, and four microphones. It also contains signal processing hardware that is able to interpret all of the data arriving from the input signals. By combining the output of these sensors, the system can follow and recognize objects in front of it, determine the direction of sound signals, and isolate them from background noise.

The parameters of the motion detection and recognition system are created using the OpenCV library. The Kinect V2 system records images which are then processed further to position the patient within the environment, and the images are shown in a video format. To implement the rehabilitation training system, the Kinect V2 SDK and OpenCV need to be configured for the VS2015 environment using C++. Furthermore, VS2015 uses the <Kinect.h> library to isolate, process, and show information about the human bones and joints. This information is shown numerically, in the form of three-dimensional coordinates, making it easy to calculate the length of the bones and the angles between them.

In our system, the functions of the Microsoft Kinect V2 and the OpenCV libraries are different but complementary. The Kinect library is used to measure and process 3D skeletal data. Also, this library provides a high-level application programming interface (API) for tracking human body movements and determining joint positions. In this way, key data about joint movements and positions is obtained. In our system, OpenCV is used to process and display two-dimensional video data streams captured by Kinect; for example, OpenCV is used to display, in real time, a video stream and the skeletal data obtained from Kinect onto a computer screen in order to visualize human posture.

#### 4.2.2. Rehabilitation Training System Components and Operating Principles

**Figure 4:**
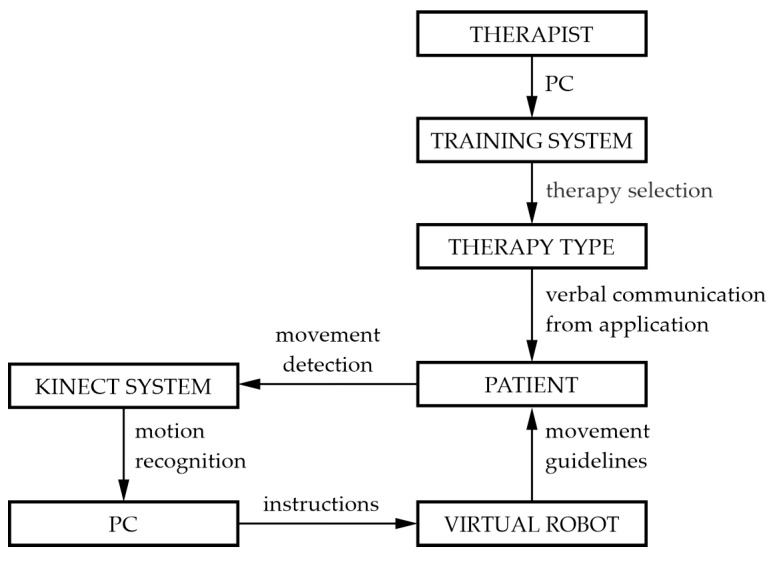
Rehabilitation training system architecture.

The computer is equipped with CoppeliaSim 4.0 Edu scene recognition software. This software contains a virtual humanoid robot also designed for human–robot interaction. Furthermore, the software is able to use verbal commands to communicate with the patient, and these can be used to direct the patient in the realization of different movements during the rehabilitation session. At the same time, the virtual humanoid robot demonstrates movements prescribed by the therapy, which the patient needs to repeat. The rehabilitation training system uses the Kinect system to detect the movement of the patient. The computer receives signals about the position of the patient’s joints in real time from the Kinect system and processes these through the software application. Figure 4 shows the architecture of the rehabilitation training system.

Human–computer interface technology was adopted to direct the patient through rehabilitation, while the virtual humanoid robot, developed in CoppeliaSim, was added to the rehabilitation system, in some therapy segments, to assume the role of the therapist in traditional rehabilitation systems.

The realization of the human–robot interaction is achieved using the virtual robot and verbal commands to instruct the patient on the movements they need to accomplish—output instructions—while the actions of the patient—input signals—are detected using the Kinect hardware for the human–computer interface. The image collection and processing of the patient is performed by Kinect to obtain the position of the segments, i.e., bones. The obtained bone positions are processed by Open CV, after which the bones are drawn in the form of levers (see Figure 3). Based on the obtained data, the lengths of the limbs and the angles in the joints are calculated, which is how the recognition of the patient’s position and movement is achieved. The rehabilitation session begins and unfolds as the virtual humanoid robot communicates with the patient.

#### 4.2.3. Modules

The rehabilitation training system consists of four modules: (i) the Human–Robot Interaction (HRI) module, (ii) the Human Motion Perception (HMP) module, (iii) the Human–Computer Interaction (HCI) module, and (iv) the Interactive Scenes (IS) module.

**Figure 5:**
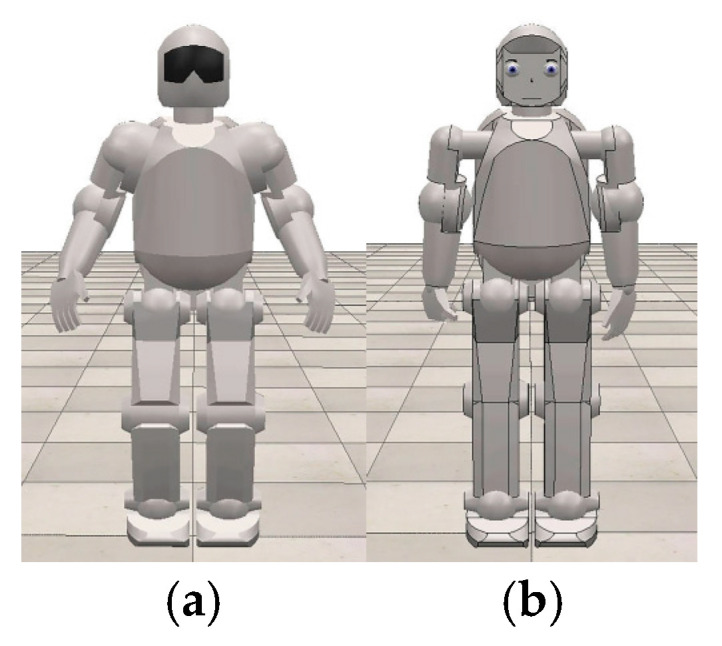
A comparison of the virtual robots: (**a**) Asti robot; (**b**) modified rehabilitation robot.

***HRI module***. Based on the existing robot Asti (Figure 5a), downloaded from the CoppeliaSim mobile robot database, a new virtual humanoid robot was developed to meet the needs of rehabilitation. The Asti robot has arms with a total of six DOFs, where each arm has two DOFs shoulder and one DOF elbow, while the hands and fingers are not actuated. Further, the fingers do not have joints at all. Although this robot has a moveable neck and legs, since these segments have no significance for further research in this case, they were disregarded. It should be noted that the Asti has a rigid face which is unable to generate facial expressions, i.e., it cannot emote. The modified virtual humanoid robot (Figure 5b) has arms with a total of 14 DOFs and hands with a total of 10 DOFs. Each arm has three DOFs shoulder, two DOFs elbow, and two DOFs wrist, while each hand has five DOFs, and each finger is actuated independently, so different movements prescribed by the therapy can be realized. The modified virtual robot also has a face displaying characteristic parts—eyes, eyebrows, and a mouth—to be able to express emotions according to situational context.

**Figure 6:**
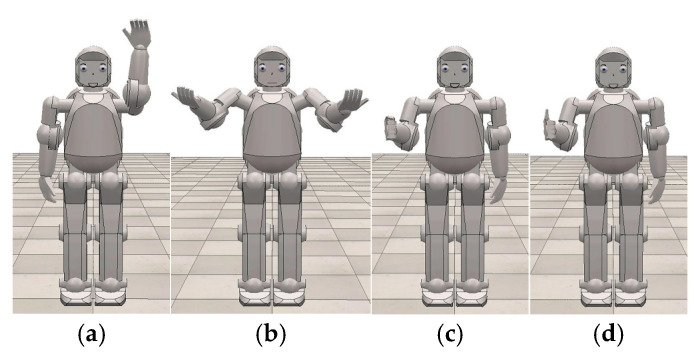
Movements of the virtual humanoid robot and their non-verbal context: (**a**) hello; (**b**) helpless shrug movement; (**c**) encouragement; (**d**) praise.

Based on available examples already in use, a virtual robot can increase the interest of children with CP in their rehabilitation exercises and can motivate and encourage them to execute the prescribed therapy movements. The modified humanoid virtual robot shown in Figure 5b can communicate verbally—with vocal commands—as well as non-verbally—using its face and upper extremities. Figure 6 shows the different movements this robot can execute, as well as their non-verbal context.

***HMP module***. The Kinect V2 can detect the 25 main joint points and is able to recognize the segments of the human body; according to this information, it can create an image of the human skeleton. Keeping in mind the end positions of the joints during helpless shrug motion execution, the Kinect system collects data on the position of the key joints of the human body. Based on this data, the positions of the joints, the angles between the bones, and the lengths of the segments are obtained. The linear kernel function in support vector machine (SVM) is used to classify the data, as well as to recognize the helpless shrug movement. After a number of tests, the accuracy of the system in recognizing the helpless shrug movement reached 96%, which satisfies the accuracy threshold for the motion recognition system.

**Figure 7:**
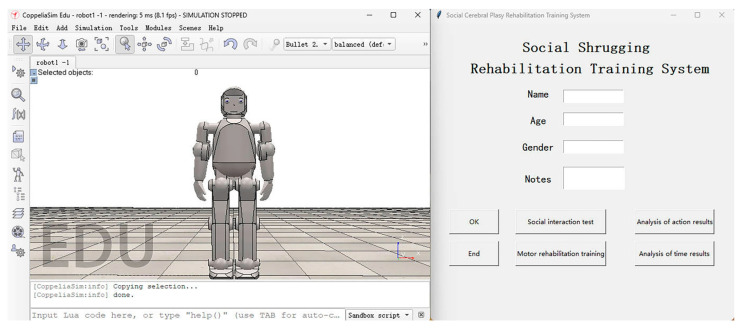
User interface display.

***HCI module***. This module is written in the Python programming language. By using a simple interactive interface (Figure 7), the therapist can easily input and save patient data and then initiate the appropriate rehabilitation regiment. After the session, the therapist receives an interpretation of the results in graph form.

**Figure 8:**
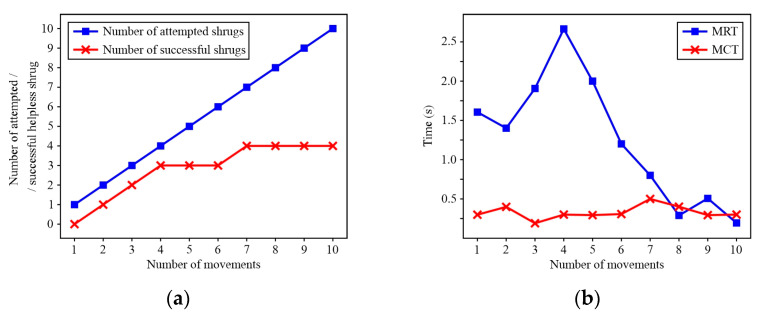
Graphic interpretation of the results: (**a**) number of attempted helpless shrugs/number of successful helpless shrugs; (**b**) movement reaction time (MRT)/movement completion time (MCT).

Before beginning the rehabilitation session, the therapist inputs the personal data for the child with CP into the user interface. By pressing the OK button, the data are saved. The regiment selection is achieved by pressing one of two buttons: “social interaction test” or “motor rehabilitation training”. After the session is finished, the system automatically analyses the reaction time of the child, the length of the session, and the results of the movement execution. By pressing the appropriate button, the therapist obtains the results in the form of graphs (Figure 8), which offer insight into all of the data pertaining to the status of the session in one location.

***IS module***. The designing of social scenarios is an important part of this observational study. Social interaction training sessions for children with CP consist of a verbal question (the robot) and a non-verbal answer (the patient). During these sessions, instead of via the guidance of a therapist, the virtual robot helps the patient to gradually master the required movements. The patient stands within the testing area during the experiment, which represents the workspace of the Kinect device.

#### 4.2.4. Summary

The rehabilitation training system is a software platform that, with the usage of the Kinect system, collects data from the coordinates of the joints of children with CP. The proposed software platform includes a virtual robot that, with verbal and non-verbal messages, directs the child to perform the required movement during re/habilitation. By applying two types of therapy—the social interaction test and motor rehabilitation training, designed for the re/habilitation of children with CP using the rehabilitation training system—results are obtained for each patient individually. At the same time, the results are stored in the database. Within the proposed system, after the test, data are obtained in the form of graphics and/or text. By analysing these data, therapists can follow the progress of each patient individually. It should be noted that the Kinect system was used because it is commercially available, which is very important from the point of view of implementation, realization, and use in medicine, enabling the very precise detection of movements of multiple joints simultaneously. In addition, the Kinect system includes integrated hardware and software for image detection and processing, enabling its ease of use by medical personnel, which is essential.

## 5. Efficiency of the Rehabilitation Training System

In this section, two types of social interaction scenarios were designed. To test the efficiency of the rehabilitation training system, an observational study was conducted with two children with CP, and the results of that study are included.

### 5.1. Scenario Design

Within the rehabilitation training system, two social interaction scenarios were designed: (i) the social interaction test and (ii) motor rehabilitation training. In both cases, the virtual humanoid robot was used instead of a therapist. The virtual robot asks questions and directs the movement of the child to help them gradually learn and master the helpless shoulder shrug movement. The child also stands in front of the Kinect device at a distance of 2 m so the device can perform the movement detection and recognition.

#### 5.1.1. Social Interaction Test

**Figure 9:**
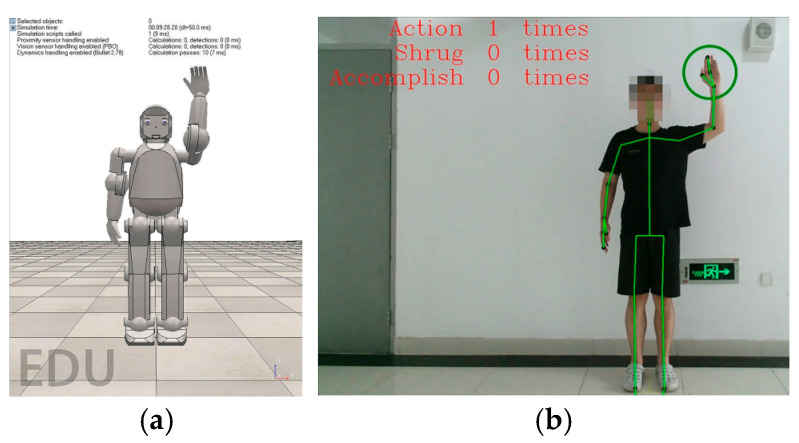
Non-verbal motion hello: (**a**) virtual robot; (**b**) patient.

In the following section, the patient is a child with CP. First, the child should be positioned in a fixed place at the test site so the rehabilitation training can be conducted. After initializing the system, the therapist starts the application and inputs the child’s data into the user interface. The system then starts the “hello” voice action, and at the same time, non-verbally communicates; specifically, the virtual robot realizes the hello motion by raising its arm up with an open hand, as can be seen in Figure 9. The child needs to answer with the same arm movement, which the system registers as the child being ready to cooperate, and the testing begins.

**Figure 10:**
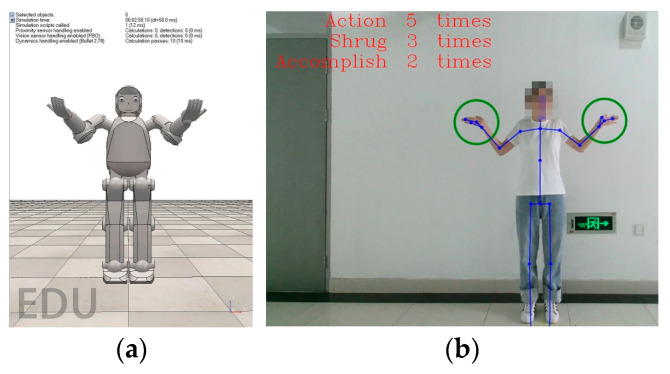
Non-verbal helpless shrug movement: (**a**) virtual robot; (**b**) patient.

As the next step, by activating the button labelled “social interaction test”, the therapist chooses the type of rehabilitation training and starts the subprogram for the patient’s rehabilitation. The virtual robot should then ask the child a question (Q: Can you solve the Rubik’s cube?), after which the child needs to answer non-verbally by shrugging their shoulders. However, if the system does not detect any kind of movement from the child within 5 s, then the verbal command repeats, and the virtual robot simultaneously shrugs its shoulders, prompting the child to repeat the movement, as shown in Figure 10.

**Figure 11:**
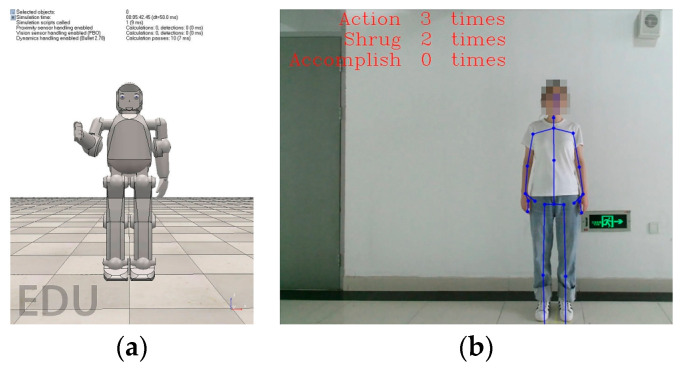
Non-verbal encourage movement: (**a**) virtual robot; (**b**) patient.

**Figure 12:**
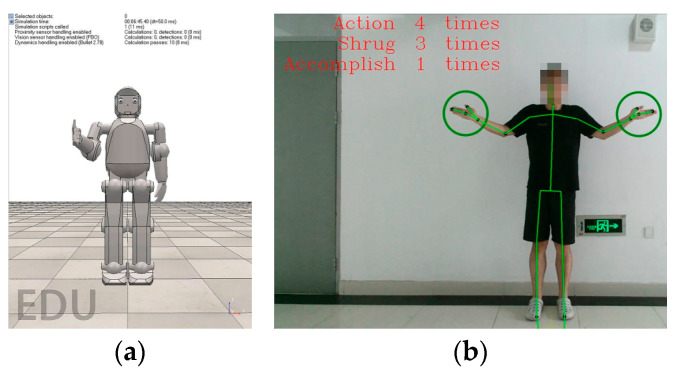
Non-verbal praise movement: (**a**) virtual robot; (**b**) patient.

The robot repeats this movement three times every time, and the system records whether the child properly repeated the movement. If the child did not execute any movements, the robot first encourages them verbally, as well as non-verbally by squeezing its fist, i.e., the encourage movement, which is shown in Figure 11, after which the robot repeats the shrug movement. If the child does execute the helpless shrug movement, the robot praises them verbally, as well as non-verbally by giving them a thumbs-up, i.e., the praise movement, as shown in Figure 12.

**Figure 13:**
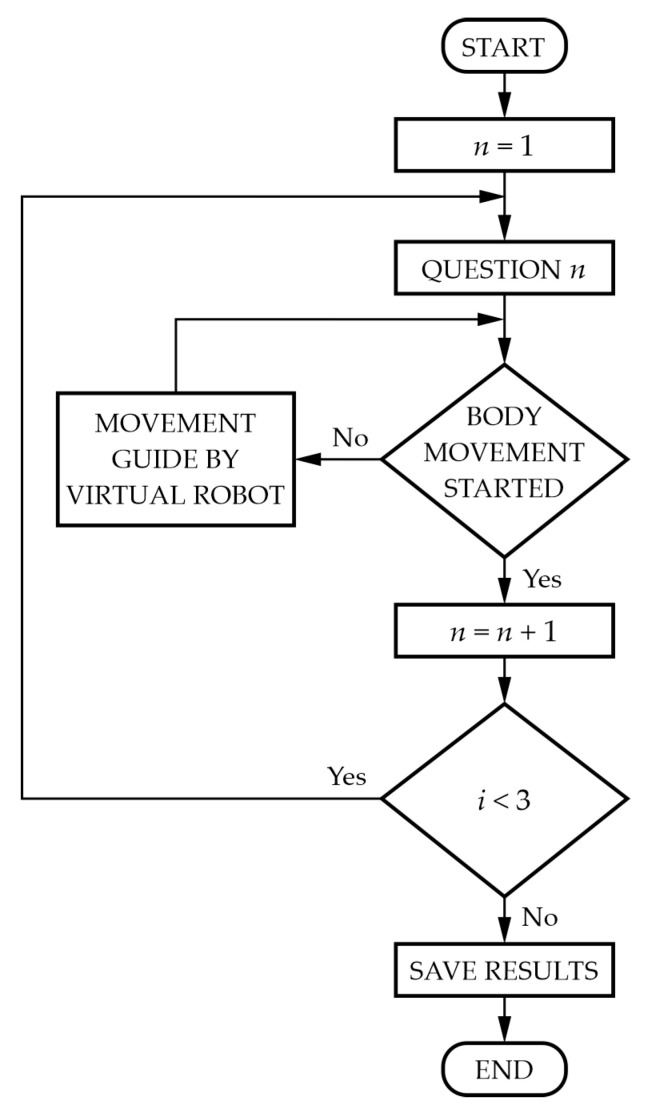
Social interaction test algorithm.

Finally, the number of successfully executed helpless shrug movements, as well as the time it took the child to perform the movement, is recorded in the form of a text file to enable the evaluation of different sessions. Figure 13 shows the social interaction test algorithm. The main goal of this test is to ascertain whether the child with CP can interact with the proposed rehabilitation system, as well as whether the child is able to realize the helpless shrug movement. If the test is unsuccessful three times in a row, showing that the patient cannot interact with the rehabilitation system, the therapist will end the test and then conduct the motor rehabilitation training, which is explained in the next section.

#### 5.1.2. Motor Rehabilitation Training

This training is designed as part of the rehabilitation training for children with CP who are unable to realize the helpless shrug movement in the context of social interaction. The system asks the child four questions (Q1: Can ants move elephants? Q2: The hammer hit the egg—why didn’t the egg crack? Q3: Why did the cat run away when it saw the mouse? Q4: Why don’t fish drown in water?) that randomly repeat for a total of ten times, as a way to practice the helpless shrug movement. The training is conducted as follows: The child is positioned on the test site, after which the therapist activates the button labelled “motor rehabilitation training” and then chooses the subprogram for practicing gross motor movement. The virtual robot first asks the child a verbal question, while simultaneously offering him a non-verbal answer by performing the helpless shrug movement, prompting the child to execute the movement as well, meaning that the child should non-verbally answer the robot’s question by mimicking the helpless shrug movement as closely to the robot’s example as possible. If the system detects movement from the child that is not the helpless shrug movement, the robot will encourage the child verbally as well as non-verbally, as is shown in Figure 11. Afterwards, the robot will ask the child a new question while again generating the helpless shrug movement. When the system detects that the movement of the child matches the helpless shrug movement, the virtual robot will praise the child verbally, as well as non-verbally by using the praise motion, as shown in Figure 12. Finally, the number of successfully executed helpless shrug movements is recorded in a text file, and the kinesitherapy is complete. The goal of the gross motor function training is for the child to master the helpless shrug movement so they can achieve better results in the social interaction test.

### 5.2. Observational Study

To justify the sample size, a number of papers dealing with similar topics were analysed. A robotic lower extremity exoskeleton used in a non-ambulatory child with CP is shown in Ref. [45]; during a study lasting 3 months, the efficiency of the Trexo Home robotic gait trainer for lower extremities with a 7-year-old girl with CP was tested. The development of a telerobotics-assisted platform for enhancing interaction with physical environments for people living with CP is shown in Ref. [46]. The platform consists of the user-side two DOFs robot Kuanser for the rehabilitation of upper extremities, the task-side Fantom Premium robot, and a virtual assistive algorithm; the efficiency of this platform was first tested on a healthy person, and then afterwards, on a child with CP. The use of customizable games for stroke rehabilitation is shown in Ref. [47]. The hardware consists of a Nintendo Wii remote and a webcam, while the software was developed using Java (games for rehabilitation) and the LookingGlass code base with a library of 3D models and scenes; the efficiency of the described system was tested on four adults that had previously experienced strokes.

The efficiency of the rehabilitation training system was tested in the Rehabilitation Services Centre in the city of Changshu with two patients, i.e., children with CP with different levels of disability. Patient A is a boy with normal intellectual ability, verbal cognitive ability, communication-related speech impediments, and the ability to learn and execute simple body movements—GMFCS scale, level I, and level III according to CFCS scale. Patient B is a girl with the following disabilities: intellectual disability, the ability to recognize simple social language, inability to use speech to communicate, partial motor function issues, as well as the inability to execute social movements—GMFCS scale, level I, and level V, according to the CFCS scale. Additionally, the inclusion criteria for children with CP are as follows: (i) GMFCS scale level I, who understand the set motor task, and (ii) CFCS scale levels I–V. The exclusion criteria for children with CP include the following: GMFCS scale levels II–V who do not understand the set motor task.

**Figure 14:**
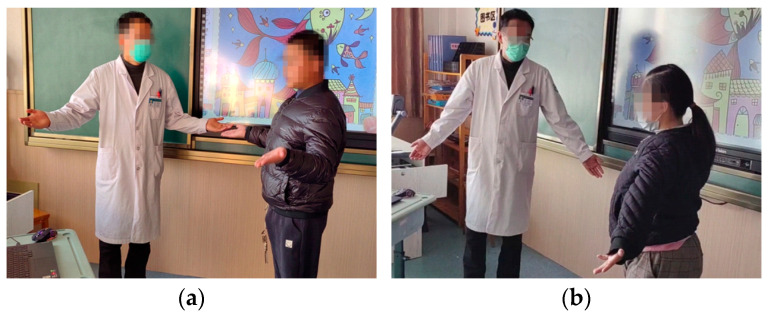
Instruction scene with therapist: (**a**) Patient A; (**b**) Patient B.

The rehabilitation of both patients consists of 5 training sessions, with each training session comprising 10 sets, with a 10 min break after every two sessions. Aside from testing the system, the goal of the rehabilitation training is for the children with CP to learn the helpless shrug movement to improve their cognitive and socio-emotional abilities. Before beginning the training, the therapist introduces the rehabilitation scenario, as well as the number of sessions and the breaks between them, to the patients. The therapist first verbally informs the patients regarding the type of movement they need to execute, and then non-verbally demonstrates it, as shown in Figure 14. Afterwards, the therapist positions the child on the test site and starts the rehabilitation training system application, as well as the rehabilitation subprogram, by activating the button labelled “social interaction test”.

During the rehabilitation training session, the following is observed and noted: (i) the number of attempts when executing the helpless shrug movement, (ii) the number of successfully executed helpless shrug movements, (iii) the movement reaction time of the patient—the interval between the end of the question the robot asks and the beginning of the patient’s movement—and (iv) how long the helpless shrug movement lasts, regardless of whether it was executed correctly of not. By analysing these parameters, the therapist can assess the progress of the patient based on the number of training sessions within an appropriate time period.

Table 3 shows the results of the experiment, i.e., the number of attempts and unsuccessful attempts, as well as successful executions, of the helpless shrug movement for both patients after five rehabilitation training sessions. Patient A achieved a satisfactory level of interaction with the robot, although the rate of success in executing the helpless shrug movement was not high. However, in every subsequent session, this patient increased the number of attempts, as well as the number of successful executions, and during the last session, the patient attempted the movement 80% of the time and succeeded 50% of the time. Therefore, after a higher number of training sessions, Patient A is able to successfully execute the helpless shrug movement. However, Patient B achieved a lower level of interaction with the virtual robot, which was expected due to the level of disability, specifically, the intellectual disability and motor function issues. However, in every subsequent session, there was moderate progress in the number of attempts, and during the final session, Patient B attempted the movement 50% of the time and succeeded in executing the helpless shrug movement 20% of the time.

**Figure 15:**
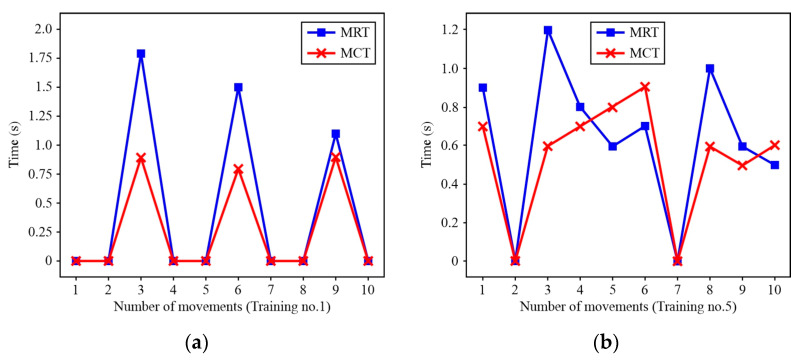
MRT/MCT for Patient A: (**a**) T1; (**b**) T5. Note: (i) value 0 means that the movement was not initiated, (ii) MCT refers to the shrug movement, regardless of whether it was successful or not.

**Figure 16:**
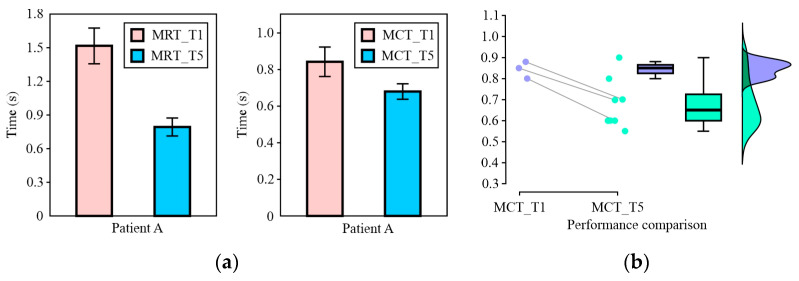
Patient A: (**a**) comparison of results, with mean and standard error estimates of MRT and MCT, at T1 (*p* = 0.093) and T5 (*p* = 0.007), respectively; (**b**) MCT results reported in raincloud plots.

Figure 15 shows the movement reaction time (MRT) and movement completion time (MCT) of Patient A. By analysing the results for Patient A after Training no. 1 (T1) and Training no. 5 (T5), it was concluded that the MRT gradually stabilized around 0.8 s. However, although there is an apparent difference in MRT, there is a statistically significant difference (Figure 16a) at a confidence level of *α* = 0.9 (*p* = 0.1). Next, according to the analysis of MCT after T5 (Figure 16b), the results show the presence of statistically significant differences (*p* = 0.007).

**Figure 17:**
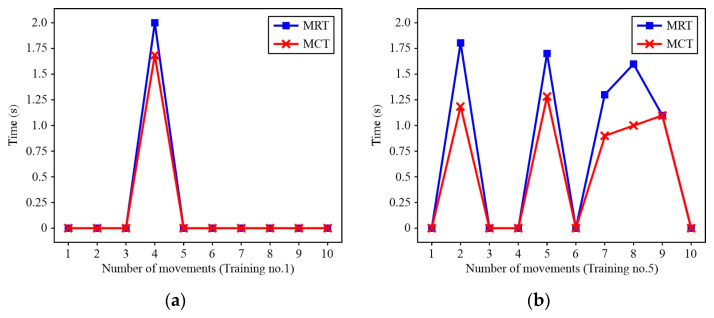
MRT/MCT for Patient B: (**a**) T1; (**b**) T5. Note: (i) value 0 means that the movement was not initiated; (ii) MCT refers to the shrug movement, regardless of whether it was successful or not; (iii) comparison could not be tested due to only one successful result at T1 (see Table 3).

According to Figure 17 (MRT and MCT of Patient B), due to both intellectual and motor function disability—difficulty in moving the limbs—managed to attempt and unsuccessfully execute only one helpless shrug movement during the first training session (see Table 3). On the other hand, after the final training session, Patient B attempted the movement five times and managed to successfully execute it once (see Table 3). Further, the MRT and MCT gradually decreased, which points to the rehabilitation training having some influence on Patient B learning and mastering the helpless shrug movement.

Based on all of the above, we can conclude that the social reaction time and mean completion time suggest the presence of an effect, ultimately providing evidence of an improvement in social interaction. The reported differences show a small effect size, and the results of the analysis are presented in Table 4.

## 6. Discussion and Conclusions

Body language is a very powerful tool in everyday communication, especially for children with non-verbal CP. Due to this, it is very important for these children to learn body language and its non-verbal context, as well as to practice non-verbal movements through rehabilitation training, which has been shown to have a positive impact on their motor function and socio-emotional skills. Since conventional therapy most often focuses on gross motor function exercises, and even the simplest ones are extremely strenuous for children, children with CP very quickly lose interest in the exercises, and the goal with this approach was to offer them a virtual robot–trainer that would make the training more fun. This is why we developed the rehabilitation training system—a hybrid robot technology for the rehabilitation of children with CP—which consists of four modules: (i) the Human–Robot Interaction (HRI) module, (ii) the Human Motion Perception (HMP) module, (iii) the Human–Computer Interaction (HCI) module, and (iv) the Interactive Scene (IS) module. Through two different training types, children with CP can gradually master social movements needed for socialization, understanding and generating the movements, along with their non-verbal contexts. The virtual humanoid robot is meant, to some extent, to replace the therapist and to motivate and encourage children with CP undergoing rehabilitation therapy. The interactive robotic system recognizes correctly executed helpless shrug movements.

The use of a physically present robot during rehabilitation requires the presence of a expert, usually an engineer, to operate the hardware (robot), as well as to make any changes to the software when adapting the therapy to each individual patient. However, a hardware failure is impossible with a virtual robot, since it does not actually exist. Also, the therapist can start the rehabilitation training system by themselves when beginning a therapy session. An additional advantage of the proposed system is the possibility of choosing the look of the virtual robot, such as using different faces, clothes, colours, scenes, and even turning the virtual robot into some kind of animal, according to the child’s preferences, which can further motivate them.

The main advantages of the rehabilitation training system include its flexibility and ease of use. Aside from its use in the rehabilitation of children with CP, the proposed system can also be used in the rehabilitation of mentally healthy people that have sustained injuries or are recovering from surgery, such as conditions involving broken bones or tendon and muscle injuries of the extremities, with minimal changes. Another advantage is that the therapist can create classifications of therapies and patients, for example, according to injury type or affected body part. Further, according to the injury type, different parameters can be observed by the system to help assess how successful the therapy was, by evaluating, e.g., the angles in the arm joints after an elbow break. These angles are already being observed by the rehabilitation training system, while recording the movements and joint positions, to form the patient’s bone positions. Therefore, the changes that would need to be made to the software are mainly related to recording the angle data for the purpose of drawing graphs and analysing the results.

Our non-contact rehabilitation technology exhibits significant differences in regards to the design concepts from those of exoskeletons and haptic robots. The presented system uses a virtual robot to guide children to imitate and complete body movements in order to achieve active rehabilitation. This method emphasizes active participation and motivates patients through gamification and an engaging design. It is particularly suitable for improving children’s autonomy in rehabilitation training. In comparison, exoskeletons and tactile robots are mainly used for mechanical assistance, and patients typically rely on machines to complete actions, with less active participation. In addition, our contactless system is based on a motion capture device—Kinect—that allows for data collection without making physical contact with the patient. This method not only improves safety, but is especially suitable for children who are sensitive to touch and avoids the psychological pressure that mechanical equipment can cause.

From an eligibility perspective, the proposed system enables the assessment of the abilities of a child with CP in real time. For example, for patients who already have some autonomy in movement, the usage of our system can further improve the accuracy and coordination of movements; for patients who have certain difficulties in performing movements, our system can effectively improve movement patterns and limb functions; and when it comes to patients with severely limited abilities, we believe that the use of mechanical aids—exoskeletons—is more beneficial for progress in rehabilitation. Our technology focuses more on improving movement control and coordination, but has certain limitations in regards to muscle strength recovery or force feedback, which is inherent in tactile robots. It should be noted that children with CP are reluctant to accept contact with mechanical devices for initiating movement during rehabilitation, as well as active muscle support and strengthening, so this is another advantage of our system, as physical contact with the proposed technology does not exist.

The efficiency of the developed rehabilitation training system was tested on two children with CP in the Rehabilitation Services Centre in the city of Changshu, China. An observational study was designed because it allows more natural real-world observation of interactions between children with CP and the virtual robot, without the interference of controlled trials. Participants are selected based on their eligibility to engage in motor exercises with verbal and non-verbal cues: Patient A (GMFCS scale, level I, and CFCS scale, level III) and Patient B (GMFCS scale, level I, and CFCS scale, level V). Although the reported cases confirm statistically significant differences for Patient A between T1 and T5 considering MCT (*p* = 0.007), the reported difference in MRT (*p* = 0.093) is at lower confidence interval, which is not always preferred. Also, providing a general conclusion about the findings is limited for the evidence due to small and limited sample size for supporting reliable and valid results. However, this certainly does not downplay the outcomes, suggesting the presence of an effect with our reported findings. The limitation of this research is the small sample size, the differing degree of disability, and differences in gender and age. Therefore, the research results cannot be generalized.

Based on the conducted observational study, the rehabilitation training system can, to some extent, have an effect on the rehabilitation of children with CP, specifically to improve the motor, social, and cognitive abilities of the child. The use of a virtual humanoid robot has a positive effect on the children’s interest in the training, which increases the number of repetitions of individual exercises, as well as the length of the therapy, which again directly strengthens the children’s musculature—the primary goal of rehabilitation. Aside from that, the children also learn to execute socially acceptable movements in the context of non-verbal interaction, which is crucial for their socialization. Finally, it should be noted that using the proposed system significantly decreases the amount of time and engagement required from the therapists, which allows them to take on more patients, which is of great importance.

In the future, we plan to increase the number of movements the virtual robot will be able to execute by using domain-specific modelling and domain-specific languages, which would allow the therapist to simply, and without the presence of a programmer, change the program, and with it, all of the aspects of the therapy being conducted. All of the analyses of the patient’s body language and uses would be described using a model, from which customized therapy could be automatically generated for patients with CP, combining verbal and non-verbal communication. This approach solves two problems that occur in robot-therapy with children with CP, i.e., (i) describing the specifics of the patient and the use of specific body language, dealing with patient variety, and (ii) the use of models for the automatic generation of therapy software and software for controlling the virtual robot.

## 7. Patents

The rehabilitation training system described in this paper currently has a patent pending.

## Figures and Tables

**Table 1:** Subjects’ responses according to the context of the verbal/non-verbal message.

Question	Shrugging Context	Verbal Communication	Non-Verbal Communication
Q1	Helplessness	15/15 (100%)	7/15 (46.7%)
Q2	Negation	15/15 (100%)	4/15 (26.7%)
Q3	Surprise	15/15 (100%)	3/15 (20.0%)
**Total**		**45/45 (100%)**	**14/45 (31.1%)**

**Table 2:** Reference positions of the arm and shoulder joints of five subjects.

Subject No.	Reference Position of the Joints							
	*α*_1_ (°)	*α*_2_ (°)	*β*_1_ (°)	*β*_2_ (°)	*γ*_1_ (°)	*γ*_2_ (°)	*h*_1_ (mm)	*h*_2_ (mm)
S1	71.3	72.2	147.9	142.3	122.7	122.4	120.3	115.3
S2	74.1	70.8	146.1	146.3	115.9	133.7	146.8	132.5
S3	72.2	74.1	148.4	146.3	112.8	120.6	131.1	127.2
S4	78.8	79.9	145.3	158.9	107.8	106.3	137.2	132.5
S5	76.1	75.1	149.7	147.5	121.8	121.3	144.1	123.5
**Total** *	74.5 ± 3.0	74.4 ± 3.5	147.5 ± 1.8	148.3 ± 6.3	116.2 ± 6.2	120.9 ± 9.7	135.9 ± 10.7	126.2 ± 7.2

* The mean value and standard deviation.

**Table 3:** The success rate of the helpless shrug movement execution by patients A and B, respectively.

Training No.	Attempted Quantity		Successful Quantity	
	Patient A	Patient B	Patient A	Patient B
T1	3	1	0	0
T2	5	2	1	0
T3	6	2	2	1
T4	6	3	2	1
T5	8	5	4	1

**Table 4:** Paired samples *t*-test results.

Measure 1	Measure 2	t	df	*p*	Cohen’s *d*	SE	Lower	Upper
MRT_AT1	MRT_AT5	3.051	2	0.093	0.550	1.80	–0.226	1.326
MCT_AT1	MCT_AT5	12.16	2	0.007	0.177	0.015	0.114	0.239
MRT_BT1	MRT_BT5	NA	NA	NA	NA	NA	NA	NA
MCT_BT1	MCT_BT5	NA	NA	NA	NA	NA	NA	NA

Note: MRT—movement reaction time; MCT—movement completion time; AT1—Patient A at Training no. 1; AT5—Patient A at Training no. 5; BT1—Patient B at Training no. 1; BT5—Patient B at Training no. 5.

## Data Availability

Data are contained within the article.
